# Vitamin D supplementation during rehabilitation in COPD: a secondary analysis of a randomized trial

**DOI:** 10.1186/1465-9921-13-84

**Published:** 2012-09-25

**Authors:** Miek Hornikx, Hans Van Remoortel, An Lehouck, Chantal Mathieu, Karen Maes, Ghislaine Gayan-Ramirez, Marc Decramer, Thierry Troosters, Wim Janssens

**Affiliations:** 1 Respiratory Division and Rehabilitation, Laboratory of Pneumology, University Hospital Gasthuisberg, KULeuven, Herestraat 49, Leuven, 3000, Belgium; 2 Division of Endocrinology, University Hospital, KULeuven, Leuven, Belgium; 3 Department of Rehabilitation Sciences, Faculty of Kinesiology and Rehabilitation Sciences, KULeuven, Leuven, Belgium

**Keywords:** Chronic obstructive pulmonary disease, Exercise capacity, Skeletal muscle, Systemic consequences, Vitamin D

## Abstract

**Rationale:**

Pulmonary rehabilitation is an important treatment for patients with Chronic Obstructive Pulmonary Disease, who are often vitamin D deficient. As vitamin D status is linked to skeletal muscle function, we aimed to explore if high dose vitamin D supplementation can improve the outcomes of rehabilitation in Chronic Obstructive Pulmonary Disease.

**Material and methods:**

This study is a post-hoc subgroup analysis of a larger randomized trial comparing a monthly dose of 100.000 IU of vitamin D with placebo to reduce exacerbations. 50 Subjects who followed a rehabilitation program during the trial are included in this analysis. We report changes from baseline in muscle strength and exercise performance between both study arms after 3 months of rehabilitation.

**Results:**

Vitamin D intervention resulted in significantly higher median vitamin D levels compared to placebo (51 [44-62] ng/ml vs 15 [13-30] ng/ml; p < 0.001). Patients receiving vitamin D had significantly larger improvements in inspiratory muscle strength (-11±12 cmH2O vs 0±14 cmH2O; p = 0.004) and maximal oxygen uptake (110±211 ml/min vs -20±187 ml/min; p = 0.029). Improvements in quadriceps strength (15±16 Nm) or six minutes walking distance (40±55 meter) were not significantly different from the effects in the placebo group (7±19 Nm and 11±74 meter; p>0.050).

**Conclusion:**

High dose vitamin D supplementation during rehabilitation may have mild additional benefits to training.

## Introduction

Chronic Obstructive Pulmonary Disease (COPD) is currently appreciated as a complex disease characterized by pulmonary and extra-pulmonary manifestations [1,2]. Among its co-morbidities or systemic consequences, skeletal muscle weakness is highly prevalent and one of the main reasons for referral to pulmonary rehabilitation [3]. Pulmonary rehabilitation programs have proven their effect in tackling muscle dysfunction. Resistance training and aerobic training have shown to enhance skeletal muscle strength, but still a large variability in training response remains and predictive factors for success are poorly understood [4-6].

Vitamin D is essential for maintaining skeletal health and low vitamin D serum (25-OHD) levels have been associated with reduced skeletal muscle strength and increased risk of falls [7-9]. In elderly individuals, vitamin D status is associated to physical performance and subsequent functional decline during long-term follow-up [10]. Randomized trials and meta-analyses in elderly systematically demonstrate that vitamin D supplementation improves balance and reduces falls by approximately 20% [11,12]. Data are less consistent in showing direct effects of vitamin D on skeletal muscle strength. According to a recent meta-analysis benefits of supplementation were only present when baseline 25-OHD levels are very low (<10ng/ml) [13]. Nevertheless, because large cross-sectional data suggests that muscle strength continues to increase from 25-OHD levels of 9ng/ml to 37ng/ml [7], it can be speculated that beneficial effects on the muscle are only seen when higher doses of supplementation are given [14].

Vitamin D deficiency is highly prevalent in COPD and the prevalence increases with disease severity [15]. Although a direct relationship between 25-OHD levels and quadriceps function has not been shown in COPD, genetic polymorphisms in the vitamin D receptor (VDR) have been associated with quadriceps strength [16,17]. Several authors have also linked vitamin D deficiency to impaired exercise capacity, a higher risk of drop-out from rehabilitation and a tendency towards reduced training benefits [17-19]. To our best knowledge, no randomized trials are currently available that have explored the effect of vitamin D supplementation on top of an exercise training program in COPD. We hypothesized that high dose vitamin D supplementation may improve the outcomes of a pulmonary rehabilitation program in patients with severe COPD [20].

The present study is an exploratory post-hoc analysis of a double-blind randomized placebo controlled trial administering a monthly dose of 100.000 IU of vitamin D to reduce exacerbations in patients with moderate to severe COPD over 1 year [21]. As part of their standard care, a subgroup was willing to participate in a 3 month rehabilitation program at the moment of randomization. The current subgroup analysis compares benefits of vitamin D supplementation with placebo on outcomes of 3 months pulmonary rehabilitation.

## Materials and methods

### Subjects and design

The present study is a post-hoc subgroup analysis of a double blind randomized controlled trial. 182 patients were randomly allocated to a monthly dose of vitamin D (100.000 IU) or a placebo and were followed for 1 year. Inclusion and exclusion criteria, study design and results have been previously described [21]. Briefly, eligible patients were current or former smokers, aged above 50 years, with a diagnosis of COPD according to the “Global Initiative for Chronic Obstructive Lung Disease” definition (post-bronchodilator Forced Expiratory Volume in one second (FEV_1_)/Forced Vital Capacity (FRC) ratio of less than 0.7) and with an FEV_1_ of less than 80% of the predicted value [22]. Patients were excluded if there was a history of hypercalcaemia, sarcoidosis or active cancer. Patients were screened during hospitalization for exacerbation and randomized 5 to 6 weeks after discharge if returned to re-convalescent state. At randomization, patients were tested in terms of muscle strength, exercise performance and health-related quality of life. If clinically indicated (symptoms of dyspnea and one of the following criteria: maximal workload (W_max_) < 90 watt, six minutes walking distance (6MWD) < 70% of predicted values, quadriceps strength (QF) < 70% of predicted values), patients were informed about outpatient pulmonary rehabilitation. 50 patients out of 182 patients were willing to participate in the training program, which started at the beginning of the trial and continued for at least 3 months. More specifically, some patients received both vitamin D (100.000 IU of cholecalciferol) and respiratory rehabilitation whereas others were treated with placebo (4 ml of arachidis oleum, vehicle) during respiratory rehabilitation. Changes over 3 months in peripheral and respiratory muscle strength, functional and maximal exercise capacity and health-related quality of life were compared between the placebo and vitamin D arm in this subgroup of 50 patients. FigureÂ 1 provides the consort flow chart. The study was approved by the Ethics committee of the university hospital of Leuven and all subjects signed written informed consent. The study was registered at clinicaltrials.gov (Clinical Trial Number: NCT00666367). 

**Figure 1:**
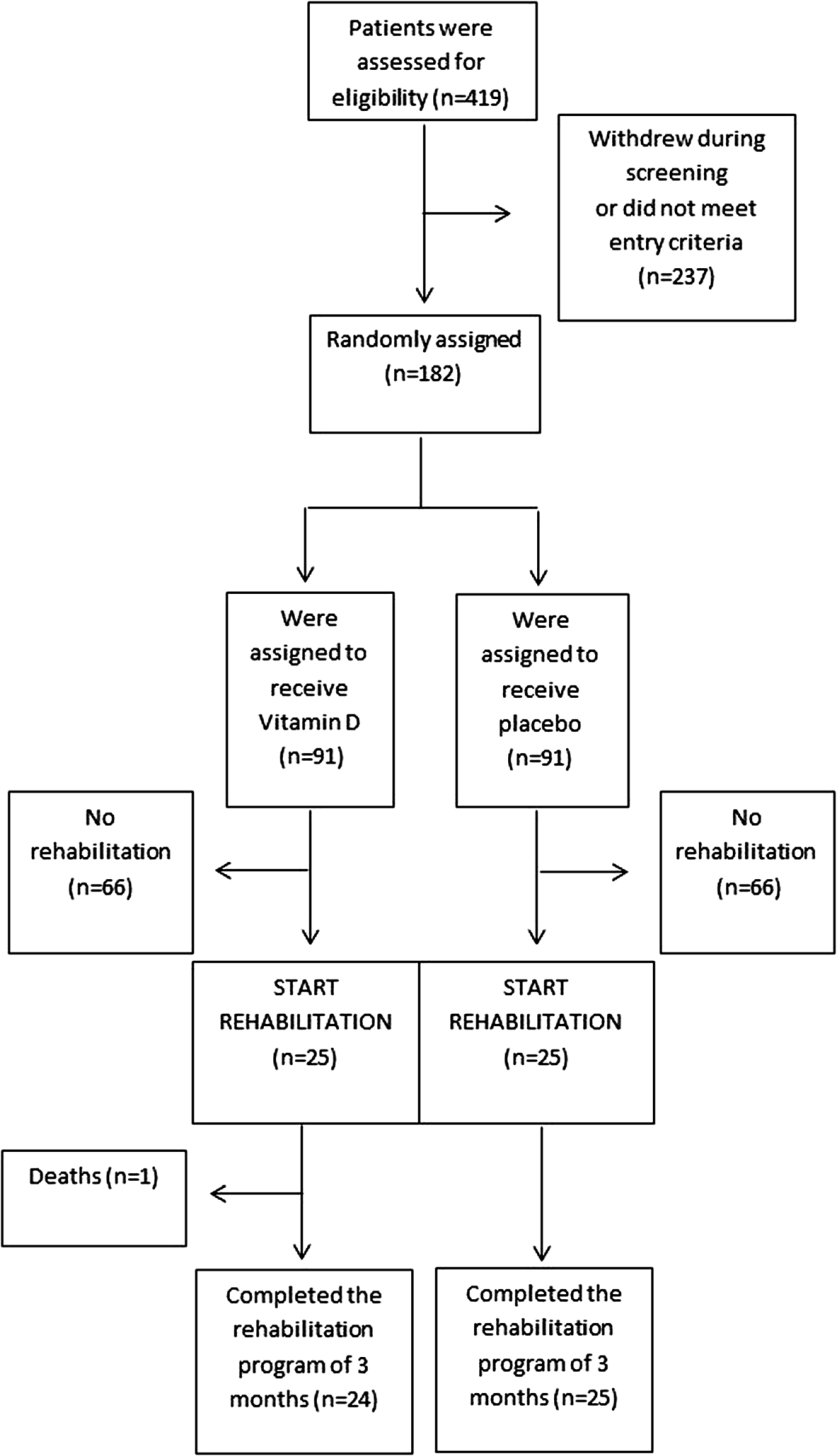
Flow chart of the study.

### Pulmonary rehabilitation program

The patients participated in a 3 months outpatient multidisciplinary rehabilitation program at a frequency of 3 times a week as previously described [23,24]. During 90 min of training, study subjects had to go through a circuit of exercises: cycling, walking on the treadmill, stair climbing, strength exercises for the upper and lower extremities and arm cranking. The initial training intensity was set at 60% of the baseline maximal workload for cycling and at 75% of the average walking speed during the baseline six minutes walking distance for treadmill walking. Physiotherapists increased patients’ workload on a weekly basis, guided by Borg-symptom scores. A Borg score of 4-6 for dyspnea or fatigue was set as a target. For the cycling training, the aim was to achieve 85% of the baseline maximal workload for 16 min, whereas for the treadmill the aim was to achieve 110% of the walking speed of the baseline six minutes walking distance for 16 min. In the strength training, patients performed 3 times of 8 repetitions with the initial load determined as 70% of the one repetition maximum (1RM) (the maximum load which can be moved only once over the full range of motion without compensatory movements). For this modality, the aim was to increase the load by 3% to 6% of the 1RM per week in order to achieve 121% of the 1RM at 3 months of training. Arm cranking was performed in 2-min blocks (1 to 3 sets) with the load set in an individual basis. Stair climbing was performed in a two-step stairs in which patients stepped up and down during 1-3 min blocks (1 to 3 sets) [23].

### Methods

#### 25-OHD level

Blood samples were taken in the fasted state at baseline prior to the study drug intake and after 3 months. Serum total 25-OHD was measured in multiple batches by radioimmunoassay (DiaSorin, Minnesota, USA) in all study participants. Total 25-OHD measures are mean values of duplicate measures referred to appropriate positive controls. Levels are expressed in ng/ml (conversion factor for nmol/l; = 2.5). Vitamin D deficiency was defined as levels below 20 ng/ml.

#### Pulmonary function

All subjects performed spirometry (Jaeger Master Screen Body; CareFusion; Germany) to determine FEV_1_ and FVC, according to European Respiratory Society recommendations [23]. The results are referred to the predicted values reported by Quanjer et al. [25].

#### Peripheral muscle strength

Isometric quadriceps strength was measured using a dynamometer (Biodex system 4 pro; Enraf Nonius; Delft, The Netherlands). Peak extension torque was measured at the dominant side, and evaluated at 60Â° of knee flexion. After an exercise trial, tests were performed at least 3 times and the best of 2 reproducible tests was used for further analyses [23]. Reference values for the quadriceps strength were developed in our laboratory [26].

#### Respiratory muscle strength

Maximal inspiratory (MIP) and expiratory (MEP) pressures were measured according to a modification of the Black and Hyatt method [27]. The modification consisted in the use of an electronic transducer instead of an aneroid manometer. Maximal inspiratory pressure was measured from residual volume, while maximal expiratory pressure was measured from TLC. Tests were repeated at least 5 times, until 3 attempts differed by <5% [23]. The highest values were related to the reference values of Rochester and Arora [28].

#### Functional exercise capacity

Functional exercise performance was measured by six minutes walking distance test in a 53-meter corridor. Encouragement was standardized. The largest distance of 2 tests was used in the analysis [23], and normal values were those described by Troosters et al. [29].

#### Maximal exercise capacity

Maximal exercise capacity was assessed by a symptom-limited incremental cycle ergometer test (Ergoline 900, Bitz, Germany). The test was performed according to the standard of the ATS/ACCP statement on Cardiopulmonary Exercise Testing [30]. After 3 min of unloaded cycling, patients started the test with a workload of 20 watts and cycled at an incremental workload of 10 watts each minute. Peak oxygen consumption, ventilation and carbon dioxide output were measured breath by breath (Sensor Medics 6200, Bilthoven, The Netherlands). Heart rate was monitored continuously by 12 leads electrocardiogram [23]. The values of peak oxygen consumption were related to the normal values by Jones et al. [31].

#### Health-related quality of life

The Chronic Respiratory Disease Questionnaire (CRDQ) was used to assess health-related quality of life. This questionnaire scores quality of life in 4 domains (dyspnea, fatigue, emotional functioning, mastery). A total score can be obtained by summation of the above mentioned domains, with higher scores indicating better quality of life [23].

#### Statistical analysis

All statistical analyses were executed with SAS version 9 statistical package (SAS Institute, Cary, North Carolina) and GraphPad Prism 4 (GraphPad Software, San Diego, California). A Kolmogorov-Smironov test was executed to test for normality of the distributions. Data were expressed as mean Â± standard deviation (if data were normally distributed) or as median [interquartile range; IQR] (if data were not normally distributed). The level of significance was set at α = 0.05. Training benefits in the total population were examined by a paired *t*-test. The change in the vitamin D level within each subgroup was evaluated by a Wilcoxon signed-ranks test. An unpaired *t*-test was used to compare the baseline characteristics of both subgroups and the difference between 3 months changes between both subgroups.

## Results

### Baseline characteristics

TableÂ 1 is showing the baseline characteristics of the subgroup of patients referred for rehabilitation (n = 50) from the total study population (n = 182), stratified according to randomization arm. Blood samples are taken prior to any study drug intake and reflect baseline 25-OHD levels. By chance, of 50 patients referred for rehabilitation exactly 25 patients were allocated to placebo, 25 patients to vitamin D supplementation and both subgroups were matched for all baseline parameters. Quadriceps strength and maximal inspiratory muscle strength tended to be lower in the vitamin D group.

**Table 1:** Baseline characteristics of patients with COPD referred for rehabilitation

	**Placebo**		**Vitamin D**		**P-value**
	**(n = 25)**		**(n = 25)**		
*Antropometric data:*					
Age (years)	69±6		67±8		0.464
Gender (male/female)	19/6		19/6		1.000
BMI (kg/m^2^)	24±6		25±5		0.587
GOLD Stages					0.492
I (n)	0		2		
II (n)	6		7		
III (n)	15		12		
IV (n)	4		4		
*Serum vitamin D level:*					
* 25-OHD (ng/ml)*	20±11		23±15		0.492
*Pulmonary function:*					
FEV_1_(l)	1.06±0.28	40±10(*)	1.22±0.50	47±18(*)	0.115
FVC (l)	2.85±0.80	83±20(*)	2.96±0.76	89±22(*)	0.328
*Muscle function:*					
QF (Nm)	106±36	83±27(*)	109±41	75±20(*)	0.242
MIP (cmH_2_O)	−77±26	72±20(*)	−67±24	63±21(*)	0.107
MEP (cmH_2_O)	158±55	83±22(*)	152±50	80±26(*)	0.685
*Exercise Performance:*					
6MWD (meter)	422±109	70±19(*)	391±135	65±23(*)	0.435
VO_2max_ (l/min)	1.21±0.48	72±24(*)	1.15±0.43	75±38(*)	0.802
W_max_ (watt)	71±27	54±22(*)	67±29	52±25(*)	0.713
*Health-Related Quality of Life:*					
CRDQ_dyspnea _(points)	16.1±4.36		17.3±4.6		0.366
CRDQ_total_ (points)	82.6±16.0		83.1±17.4		0.721

Data are expressed as mean±SD. Values are %Pred±SD when indicated with (*). BMI = Body Mass Index; 25-OHD = Serum vitamin D level; FEV_*1*_* = Forced Expiratory Volume in one second; FVC = Forced Vital Capacity; QF=Quadriceps Strength; MIP = Maximal Inspiratory Pressure; MEP = Maximal Expiratory Pressure; 6MWD = Six Minutes Walking Distance; VO*_*2max*_* = Maximal Oxygen Uptake; W*_*max*_* = Maximal Workload; CRDQ*_*dyspnea*_* = Chronic Respiratory Disease Questionnaire (Item Dyspnea); CRDQ*_*total*_* = Total score on Chronic Respiratory Disease Questionnaire.*

### Vitamin D serum level

As expected, the median 25-OHD level significantly increased within the intervention group from 15 [13-30] ng/ml at baseline to 51 [44-62] ng/ml after 3 months of rehabilitation (p < 0.001), whereas the mean 25-OHD level remained relatively stable in the group receiving placebo (19 [13-22] ng/ml vs 18 [14-34] ng/ml; p=0.089) (FigureÂ 2). At baseline, 62% (31/50) of the patients had a 25-OHD level < 20ng/ml, 16 in the placebo group and 15 in the Vitamin D arm. After study drug intake, vitamin D deficiency disappeared in all patients in the vitamin D arm without any toxicity in terms of hypercalcaemia or hypercalciuria.

**Figure 2:**
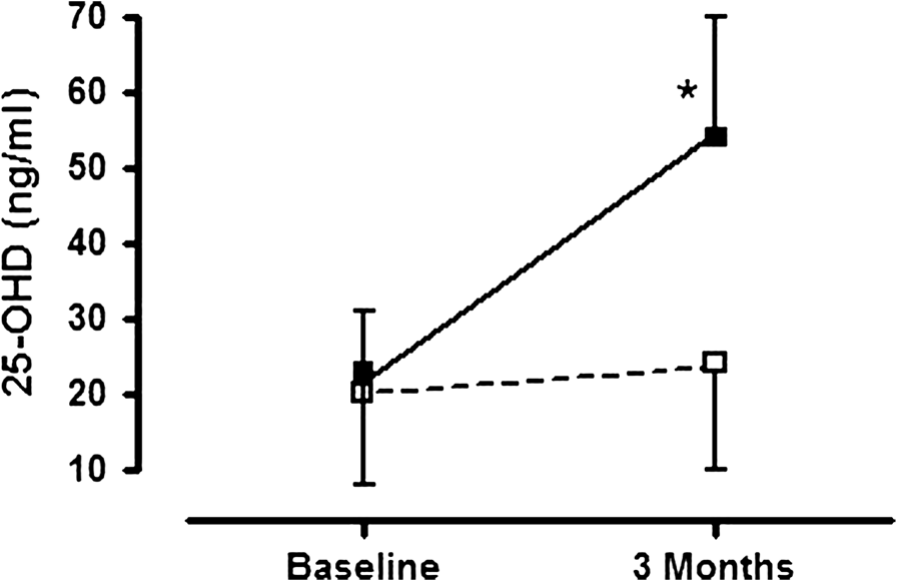
**Change in 25-OHD (Serum Vitamin D Level) in the vitamin D group (■) and in the placebo group (□).** Data are expressed as mean ± SD. Baseline results are compared with results after 3 months of rehabilitation. Results with p <0.05 are indicated with (*).

### Outcomes of rehabilitation

#### Total study population

In the total study population conventional rehabilitation during 3 months resulted in an increased muscle strength (ΔQF 11±18 Nm, p < 0.001; ΔMIP -5±14 cmH2O, p=0.01 and ΔMEP 9±37 cmH2O; p=0.105). Maximal exercise capacity tended to improve (ΔW_max_ 6±13 watt, p=0.002; ΔVO_2max_ 45±108 ml/min, p=0.144) and a statistical significant effect was found in functional exercise capacity (Δ6MWD 25±66 m, p=0.012). Quality of life scores significantly improved. The obtained effects in the total score (CRDQ_total_) and in the sub-item for dyspnea (CRDQ_dyspea_) were much larger than the respective 10 and 2.5 points increase required to obtain an minimal important clinical benefit (ΔCRDQ_total_ 15±9.3, p < 0.001; ΔCRDQ_dyspnea_ 6.0±4.6, p < 0.001).

#### Vitamin D group and placebo group

TableÂ 2 and FigureÂ 3 provide an overview of the changes in muscle strength after 3 months of pulmonary rehabilitation when comparing the vitamin arm to the placebo group. Patients receiving vitamin D had a statistical significantly higher increase in maximal inspiratory strength. Quadriceps strength and maximal expiratory strength improved more in the vitamin D group compared to the placebo group, but without reaching statistical significance. The effects were not more pronounced in vitamin D deficient patients (TableÂ 3).

**Table 2:** Changes in peripheral and respiratory muscle strength in both groups after rehabilitation

	**Placebo**	**Vit D**	**P-value**
	**(n=25)**	**(n=24)**	
Δ Muscle Function			
QF (Nm)	7±19	15±16	0.121
MIP (cmH2O)	0±14	−11±12	0.004
MEP (cmH2O)	6±41	13±35	0.511

Data are expressed as mean±SD. QF = Quadriceps Strength; MIP = Inspiratory Muscle Strength; MEP = Expiratory Muscle Strength.

**Figure 3:**
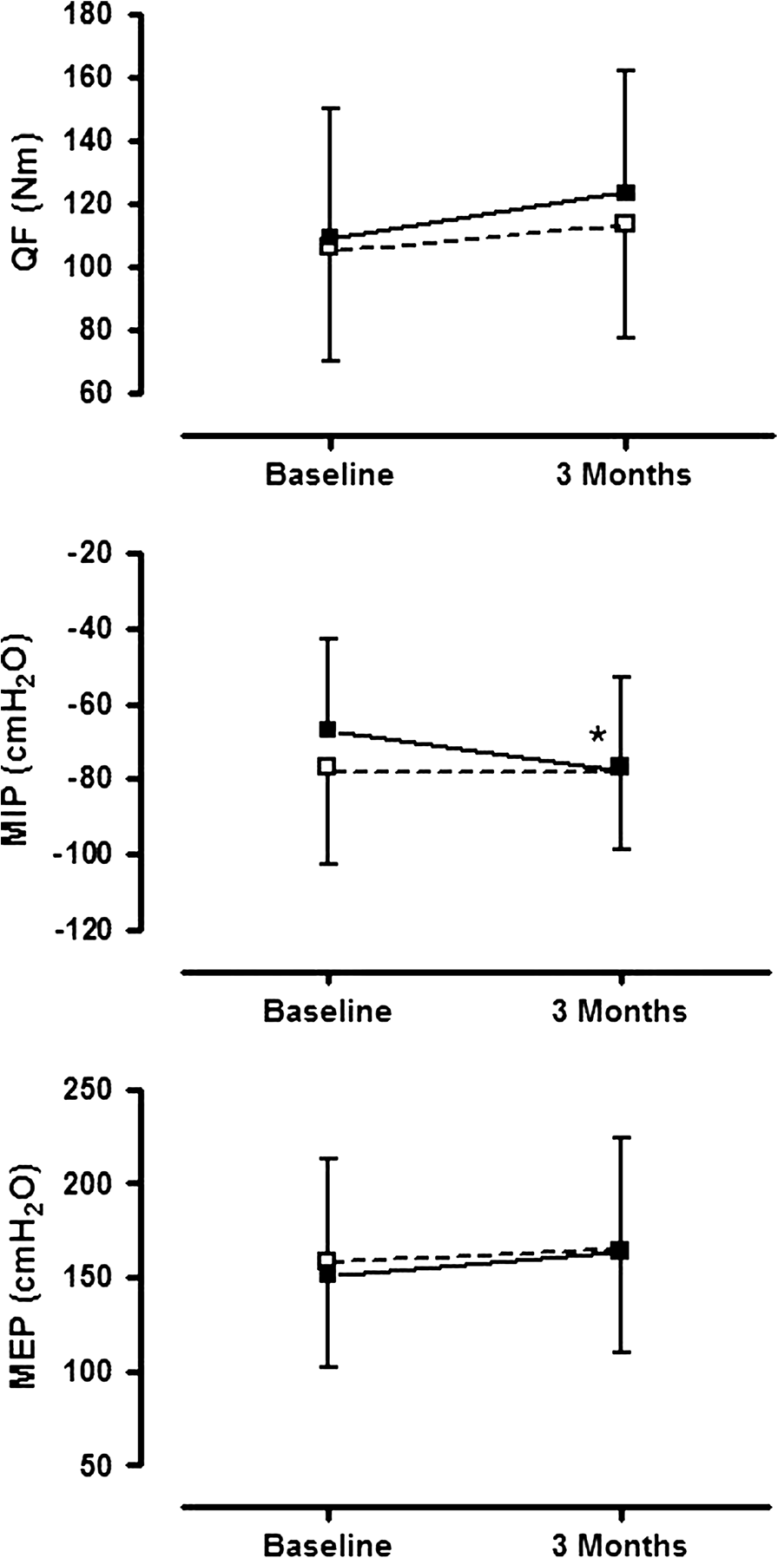
**Changes in muscle strength (QF = Quadriceps Strength; MIP = Inspiratory Muscle Strength; MEP = Expiratory Muscle Strength) in the vitamin D group (■) and in the placebo group (□).** Data are expressed as mean ± SD. Baseline results are compared with results after 3 months of rehabilitation. Results with p <0.05 are indicated with (*).

**Table 3:** Changes in peripheral and respiratory muscle strength in vitamin D deficient patients after rehabilitation

	**Placebo**	**Vit D**	**P-value**
	**(25-OHD < 20)**	**(25-OHD < 20)**	
	**(n=16)**	**(n=15)**	
Δ Muscle Function			
QF (Nm)	6±19	15±19	0.176
MIP (cmH2O)	0±16	−9±6	0.046
MEP (cmH2O)	2±44	16±33	0.333

Data are expressed as mean ± SD. QF = Quadriceps Strength; MIP = Inspiratory Muscle Strength; MEP = Expiratory Muscle Strength.

TableÂ 4 and FigureÂ 4 summarize the changes in exercise performance after 3 months of rehabilitation when comparing the vitamin D group with the placebo group. Statistically significant improvements in maximal oxygen uptake were obtained in the vitamin D group compared to placebo. Patients receiving vitamin D also improved more in terms of maximal workload and six minutes walking distance, but these effects were not statistically significant. When performing the same analyses in patients with vitamin D deficiency at baseline (TableÂ 5), no significant changes could be found. We finally compared changes in quality of life between the vitamin D group and the placebo group. We found no statistical significant difference, although a positive trend in favor of the vitamin D group was observed for the dyspnea item (Δ7.0±5.2 in the vitamin D group vs Δ5.5±3.9 in the placebo group; p=0.16)

**Table 4:** Changes in exercise performance in both groups after rehabilitation

	**Placebo**	**Vit D**	**P-value**
	**(n=25)**	**(n=24)**	
Δ Exercise Performance			
6MWD (meter)	11±74	40±55	0.130
VO_2max_ (ml/min)	−20±187	110±211	0.029
W_max_ (watt)	3±13	10±13	0.053

*Data are expressed as mean±SD. 6MWD = Six Minutes Walking Distance; VO*_*2max*_* = Maximal Oxygen Uptake; W*_*max*_* = Maximal Workload.*

**Figure 4:**
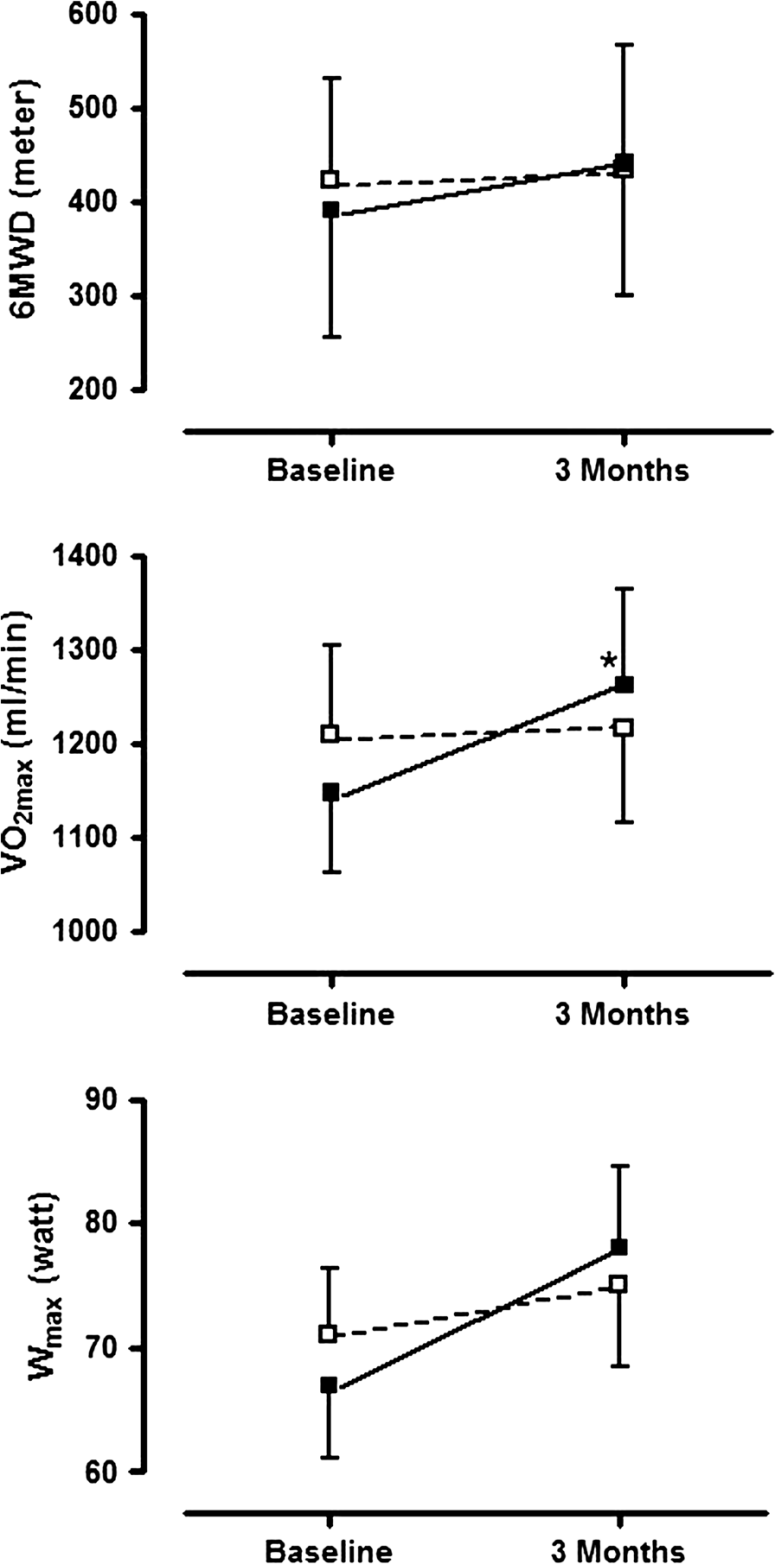
**Changes in exercise performance (6MWD = Six Minutes Walking Distance; VO**_**2max**_** = Maximal Oxygen Uptake; W**_**max**_** = Maximal Workload) in the vitamin D group (■) and in the placebo group (□).** Data are expressed as mean±SD. Baseline results are compared with results after 3 months of rehabilitation. Results with p <0.05 are indicated with (*).

**Table 5:** Changes in exercise performance in vitamin D deficient patients after rehabilitation

	**Placebo**	**Vit D**	**P-value**
	**(25-OHD < 20)**	**(25-OHD < 20)**	
	**(n=16)**	**(n=15)**	
Δ Exercise Performance			
6MWD (meter)	18±45	45±44	0.106
VO_2max_ (ml/min)	−8±190	96±202	0.156
W_max_ (watt)	3±14	10±12	0.140

*Data are expressed as mean ± SD. 6MWD = Six Minutes Walking Distance; VO*_*2max*_* = Maximal Oxygen Uptake; W*_*max*_* = Maximal Workload.*

## Discussion

The present study is the first placebo controlled intervention study exploring the effect of supplementation with high doses of vitamin D in a 3 month rehabilitation program in patients with COPD. We found that, compared to training alone, patients receiving a monthly dose of 100.000 IU of vitamin D had a larger improvement on inspiratory muscle strength and peak exercise tolerance. There was a similar trend towards higher quadriceps strength, six minutes walking distance and dyspnea scores, but these effects did not reach statistical significance.

Although the reported effects are rather limited, the current data support the hypothesis that vitamin D supplementation may enhance training effects in disabled patients with muscle weakness and reduced exercise capacity. There are two reasons which may explain why a larger and general benefit could not be found. Most importantly, our data are post-hoc subgroup analyses of a larger randomized controlled trial and therefore not designed nor sufficiently powered to demonstrate clinically significant benefits. Secondly, our training program in the total study group yielded important effects on quality of life and symptoms, but resulted in somewhat smaller effects than expected on physiological outcomes. As the latter outcomes would be preferentially affected by vitamin D because of common pathways in the muscle [8], the window for improvement under vitamin D was a priori small. The absence of clinically relevant training benefits in quadriceps strength and functional exercise capacity in the total subgroup may reflect the individual variability in training response, especially observed in very disabled COPD. Nevertheless, largest effects were preferentially obtained in the vitamin D supplemented group, whereas the placebo group seemed more resistant to physiological improvements.

Currently, it is still unclear which serum vitamin D levels are needed for an optimal muscle function [7,14,32]. Different intervention studies indicate that vitamin D supplementation may only improve muscle strength when baseline levels are deficient but surprisingly, few studies have explored this question in a context of training [33]. Bunout et al. reported that a daily intake of 400 IU of vitamin D with 800 mg of calcium plus biweekly strength, balance and aerobic exercises in an elderly population did not enhance muscle mass or function compared to either exercise or vitamin D alone [34]. A more recent randomized trial evaluated whether a daily consumption of fortified milk (800 IU of vitamin D and 1000 mg of calcium) enhanced the effects of resistance training in a community dwelling elderly population, but again no significant effect could be found [35]. Different authors therefore suggested that the supplementation dosage of vitamin D in these trials was probably insufficient to obtain potential benefits [14,33]. Our data support this idea as the high dose of vitamin D (corresponding to a daily intake of 3300 IU) increased median 25-OHD levels up-to 51 [44-62] ng/ml in the intervention arm with superior clinical effects compared to placebo. We also evaluated whether larger benefits were present in the subgroup of patients being vitamin D deficient at baseline. No significant relationship could be found but we should acknowledge that these subgroups were probably too small to appreciate any difference.

The effects of vitamin D on the skeletal muscle are either direct on contractility via blood calcium levels, but most are indirectly mediated through the vitamin D receptor (VDR) which is abundantly expressed in the skeletal muscle [36]. Vitamin D deficiency is shown to reduce actin and troponin content, to impair calcium uptake in the sarcoplasmatic reticulum, to down-regulate protein synthesis and to increase apoptosis [8,37], processes which are all described in skeletal muscles of patients with COPD [38-40]. Typically, severe vitamin D deficiency results in a type II fiber atrophy with interfibrillar spaces infiltrated with fat, fibrocytes and glycogen [35]. As skeletal muscles of patients with COPD are characterized by fiber type shifts towards a type II profile, vitamin D deficiency may have a superimposed deleterious effect [37,41]. Since patients with COPD are often vitamin D deficient and frequently suffer from skeletal muscle weakness, they compose an interesting target population for combined intervention studies with vitamin D supplements and training [42]. Currently, a randomized trial specifically designed to study these questions in COPD is ongoing (clinicaltrials.gov. NCT01416701). Our data generate aspiration that a significant and clinical benefit may be revealed.

Overall, the present post-hoc analysis supports the idea that in patients with COPD high dose supplementation with vitamin D can be beneficial when combined with exercise training. Our data should be confirmed by other studies sufficiently powered to evaluate clinical and sustained benefits in patients with COPD or specific subgroups. Only then recommendations on the appropriate use and dosage of vitamin D supplements in a clinical context of training can be made.

## Competing interests

The author(s) declare that they have no competing interests.

## Authors’ contribution

MH, HVR, MD, TT and WJ carried out the rehabilitation program. KM, GGR, MD, TT and WJ designed the study analysis. AL, CM, MD and WJ designed and carried out the randomized trial. MH, TT and WJ drafted the manuscript. All authors read and approved the final manuscript.

## Support

Grant from the Applied Biomedical Research Program, Agency for Innovation by Science and Technology (IWT-TBM: G335102)

KM, WJ and CM are supported by the research foundation Flanders (FWO).

MH is funded by the FWO Grant #G.0598.09N.
